# A 35 MHz/105 MHz Dual-Element Focused Transducer for Intravascular Ultrasound Tissue Imaging Using the Third Harmonic

**DOI:** 10.3390/s18072290

**Published:** 2018-07-15

**Authors:** Junsu Lee, Ju-Young Moon, Jin Ho Chang

**Affiliations:** 1 Department of Electronic Engineering, Sogang University, Seoul 04107, Korea; leejs@sogang.ac.kr; 2 Institute of Integrated Biotechnology, Sogang University, Seoul 04107, Korea; zachary@sogang.ac.kr; 3 Department of Biomedical Engineering, Sogang University, Seoul 04107, Korea

**Keywords:** high-frequency ultrasound transducer, intravascular ultrasound, tissue harmonic imaging, dual-frequency IVUS transducer, dual-element IVUS transducer

## Abstract

The superharmonic imaging of tissue has the potential for high spatial and contrast resolutions, compared to the fundamental and second harmonic imaging. For this technique, the spectral bandwidth of an ultrasound transducer is divided for transmission of ultrasound and reception of its superharmonics (i.e., higher than the second harmonic). Due to the spectral division for the transmission and reception, transmitted ultrasound energy is not sufficient to induce superharmonics in media without using contrast agents, and it is difficult that a transducer has a −6 dB fractional bandwidth of higher than 100%. For the superharmonic imaging of tissue, thus, multi-frequency array transducers are the best choice if available; transmit and receive elements are separate and have different center frequencies. However, the construction of a multi-frequency transducer for intravascular ultrasound (IVUS) imaging is particularly demanding because of its small size of less than 1 mm. Here, we report a recently developed dual-element focused IVUS transducer for the third harmonic imaging of tissue, which consists of a 35-MHz element for ultrasound transmission and a 105-MHz element for third harmonic reception. For high quality third harmonic imaging, both elements were fabricated to have the same focus at 2.5 mm. The results of tissue mimicking phantom tests demonstrated that the third harmonic images produced by the developed transducer had higher spatial resolution and deeper imaging depth than the fundamental images.

## 1. Introduction

Due to the nonlinear nature of biological media, the harmonic components of transmitted ultrasound are generated as the ultrasound propagates through the media [1]. The energy of harmonics is nonlinearly proportional to transmitted ultrasound pressure; for example, the second harmonic energy is determined by the square of the energy in transmitted ultrasound [2]. Therefore, the main lobe energy in the beam profile of transmitted ultrasound mainly contributes to the generation of the harmonics, thus leading to enhancing contrast resolution [3,4] because the levels of the side and grating lobes are considerably low in the harmonic beam profiles. Additionally, the main lobe width of a harmonic beam profile is narrower than that of a transmit beam profile and decreases as the harmonic order increases [5]. Due to these properties, tissue harmonic imaging is capable of providing a higher spatial resolution than fundamental imaging, and the spatial resolution improves as the harmonic order increases. For these reasons, the second harmonic imaging of tissue is generally employed in modern ultrasound imaging systems. However, the superharmonic imaging is not yet a common imaging mode because a transducer with broad spectral bandwidth is not available for transmitting ultrasound and receiving its superharmonic components (i.e., higher than the second harmonic). Interleaved dual frequency array transducers can be the solution [5,6,7], but the arrays suffer from a high level of grating lobes in fundamental imaging because the pitch of the elements generating fundamental ultrasound is doubled [8]. Note that a transducer should provide fundamental images as well as harmonic images for accurate and efficient diagnosis.

For accurate diagnosis of atherosclerosis, it is also important that intravascular ultrasound (IVUS) images have high spatial and contrast resolutions. Active inflammation, severe stenosis, blood vessel remodeling, and a thin fibrous cap with a large lipid core are indispensable indications of vulnerable plaques [9,10], but current IVUS images do not have enough spatial and contrast resolutions to accurately detect the indicators. For example, the thickness of a thin fibrous cap (<65 μm) cannot be currently measured. In addition, lipid-rich plaque and blood vessel layers cannot be clearly visualized, which may cause the inaccurate of plaque composition assessment that is closely associated with plaque vulnerability [10,11]. This is so because conventional IVUS transducers have a flat (i.e., unfocused) aperture and center frequency in range of 20–40 MHz. To overcome the problem, focused IVUS transducers with a center frequency of 45–50 MHz have recently been developed [12,13], but the relatively high frequency may not be adequate to obtain the information about the overall morphological change of blood vessels due to atherosclerosis; vessel remodeling, lumen size, and wall morphology are closely related to the atheromatous plaque burden [10]. Note that ultrasound imaging depth decreases as operating frequency increases. For this end, dual-frequency IVUS transducers are the best choice to achieve both high spatial resolution and deep imaging depth: low frequency element for general IVUS imaging and high frequency element for superficial microstructure imaging [14,15,16]. Additionally, those IVUS transducers facilitate the superharmonic imaging of tissue, thus further improving spatial and contrast resolutions. However, the structure in which two elements are attached back and forth [14,15] cannot be used for tissue harmonic imaging because the harmonics generated in tissue cannot be received. Although the arrangement of two elements side by side in the elevation direction is suitable for tissue harmonic imaging, the simple arrangement of two pre-focused elements is not the case [16]. In this case, harmonic signals cannot be received effectively because the focal positions of the two elements are different. For tissue harmonic imaging, therefore, dual-frequency elements arranged side by side in the elevation direction should have a spherical shape so that their focal positions can be the same; recently, we have reported a dual-frequency oblong-shaped-focused IVUS transducer for the second harmonic imaging of tissue [17], in which the two outer 35-MHz elements play a role of transmitters, and the center 70-MHz element serves as the receiver of the second harmonic signals.

For superharmonic imaging in conjunction with ultrasound contrast agents, dual-frequency IVUS transducers in which two elements were stacked vertically were developed [18,19,20]. The lower layer element is responsible for resonating injected microbubbles that generate superharmonics. For this, the center frequency of the lower layer is in the range of 2–10 MHz. Since the upper layer element receives the superharmonics, the center frequency is 30 MHz. These transducers may be suitable for obtaining information about the proliferation of the adventitial vasa vasorum that is closely related to early atherosclerotic plaque [21], but not for the superharmonic imaging of tissue. The lower layer has a relatively large flat aperture such as 0.6 × 3 mm^2^, so that the natural focal depth is far beyond the imaging region of interest. This means that the size and shape of the element are inappropriate for tissue imaging. Additionally, the operating frequencies are too low to acquire high quality images. If the center frequencies are increased for enhancing spatial resolution, the upper layer element causes severe distortion and attenuation of the ultrasound generated by the lower layer element. This hampers the transmission of ultrasound energy high enough to produce superharmonics within tissue.

In this paper, we report a recently developed dual-element IVUS transducer for the third harmonic imaging of tissue. The two elements are arranged side by side in the elevation direction and spherically shaped with a radius of 2.5 mm. This configuration was adopted for optimal generation and reception of harmonics as well as avoiding the misalignment of the beam profiles of the two elements in the imaging region of interest. The two elements were designed to have center frequencies of 35 MHz for ultrasound transmission and 105 MHz for the reception of the third harmonic, respectively. High efficient transmission and reception of ultrasound require electrical impedance matching between a transducer and an imaging system (or signal wires) [22]. At a given element size, the 105-MHz element inevitably has a much lower electrical impedance than the 35-MHz element if the same piezoelectric material is used [8]. To avoid this, lithium niobate (LiNbO_3_) was used for the 105-MHz element, whereas PZT-5H served as the active material of the 35-MHz element. Note that the clamped dielectric constant of LiNbO_3_ is lower than that of PZT-5H. The dual-element transducer was designed using a KLM model based simulator, and its characteristics and imaging performance were evaluated.

## 2. Design and Fabrication of Dual-Element Focused Transducers

### 2.1. Transducer Design

**Figure 1:**
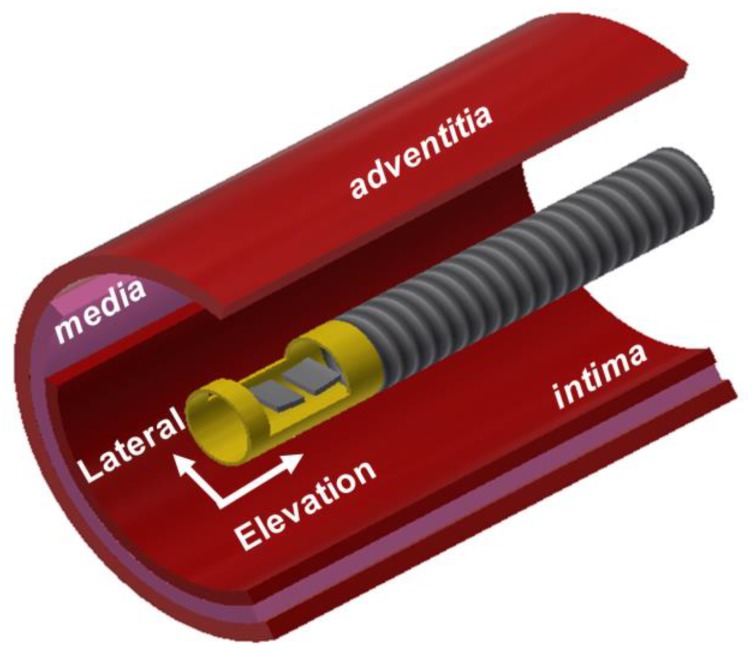
Conceptual illustration of the proposed dual-element oblong-shaped-focused transducer for intravascular ultrasound tissue imaging using the third harmonic.

The diameter of IVUS catheters containing an ultrasound transducer is commonly less than 1 mm to allow easy insertion of the catheter into blood vessels. Since aperture size is one of the parameters that determine the spatial resolution of ultrasound images, the size should be as large as possible and typically less than 0.5 mm. For this reason, the 35-MHz transmitter and 105-MHz receiver can be arranged side by side only in the elevation direction as shown in Figure 1; the size of each element was chosen to be 0.5 × 0.5 mm^2^ and kerf was 0.1 mm. In this configuration, the two elements are located outside the imaging plane formed by the lateral and axial axes. This may lead to the misalignment of the beam profiles of the two elements unless their focal depth lies on the imaging plane. For the dual-element transducer, therefore, the aperture consisting of the two elements was spherically shaped with a radius of 2.5 mm. By doing so, the focal depth of the two elements was the same and the misalignment of the two beam profiles could be minimized. This was verified through beam simulation using a Field II program.

**Figure 2:**
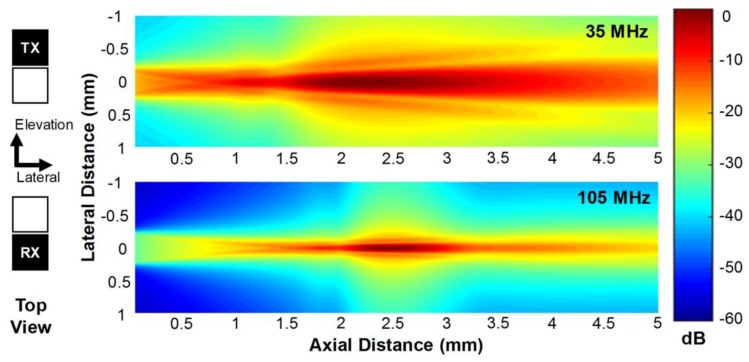
Transmit (TX) beam profile generated by the 35-MHz element (upper) and receive (RX) beam profile by the 105-MHz element (lower), which were obtained by the Field II simulation.

From the simulation, it was learned that the natural focal depth of the 35-MHz element was 1.71 mm and that of the 105-MHz element was 5.04 mm. Note that the natural focal depth of a square aperture can be calculated using 0.339 × (L^2^/λ) where L is the side length of the aperture and λ is the wavelength [1]. The geometric focus does not perfectly work for the 35-MHz element because the focal length is deeper than its natural focal depth. Nevertheless, the geometric focus considerably improves the lateral resolution of images [13]. As shown in Figure 2, the transmit (TX) beam profile generated by the 35-MHz element had the peak pressure at a depth of 2.21 mm and the depth of focus (DOF_−3dB_) in the range of 1.73 to 3.01 mm. Note that DOF_−3dB_ is defined as the −3 dB beam width in the axial direction. From this result, it was seen that the geometric focus changed the peak pressure depth from 1.71 to 2.21 mm although the geometric focal length was deeper than the natural focal depth. On the other hand, the 105-MHz element had the receive (RX) beam profile with the peak pressure at 2.45 mm and its DOF_−3dB_ ranged 2.22 to 2.76 mm, which also implies that the geometric focus worked well for the 105-MHz element because the geometric focal length was shorter than the natural focal depth. As a result, the TX and RX beams generated by the proposed dual-element focused transducer are well distributed on the imaging plane and the DOF regions overlap sufficiently to produce and receive the third harmonic signals.

**Figure 3:**
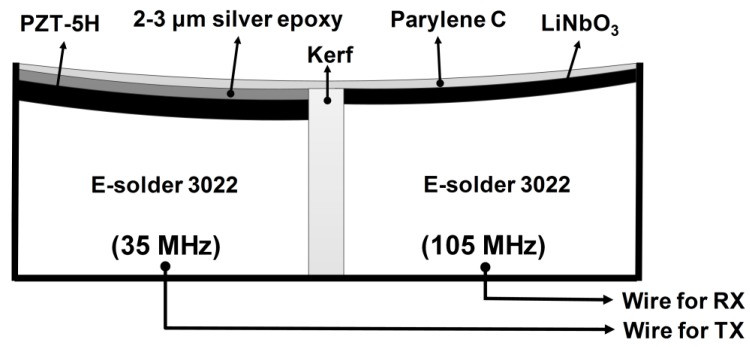
Structure of the proposed dual-element focused transducer. TX and RX stand for transmission and reception, respectively.

**Figure 4:**
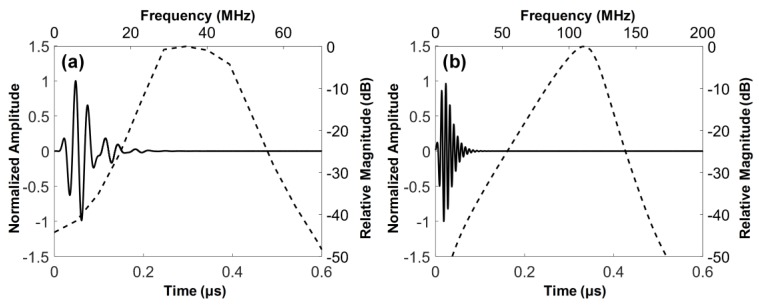
Simulated pulse-echo responses (solid line) and their frequency spectra (dashed line): (**a**) 35-MHz and (**b**) 105-MHz elements of the dual-element focused transducer.

Based on the simulation results, we designed the structure of a dual-element focused IVUS transducer as shown in Figure 3. For the 35-MHz element, PZT-5H (3203HD, CTS Technology, Sparta, IL, USA) was chosen as an active material, whereas lead-free single-crystal LiNbO_3_ (Boston Piezo-Optics, Bellingham, MA, USA) was used for the 105-MHz element. Note that LiNbO_3_ was selected to prevent the electrical impedance of the 105-MHz element from becoming too small to be driven because the clamped dielectric constant of LiNbO_3_ is lower than that of PZT-5H (i.e., 27.9 vs. 1200). As a backing layer, conductive epoxy (E-solder 3022, Von Roll USA Inc., Schenectady, NY, USA) was chosen for easy connection between the piezoelectric materials and signal wires. For the 35-MHz element, two acoustic matching layers were employed. The theoretical optimum acoustic impedances of two layers can be calculated by [23]

(1) $$Z_{m1}={\left(Z_{P}^{4}Z_{L}^{3}\right)}^{1/7}\;\mathrm{and}\;Z_{m2}={\left(Z_{P}^{1}Z_{L}^{6}\right)}^{1/7}$$

where *Z_m_*_1_ and *Z_m_*_2_ are the acoustic impedances of the first and the second matching layers, *Z_P_* and *Z_L_* are those of PZT-5H (or LiNbO_3_) and tissue (i.e., 1.5 MRayls). From Equation (1), the acoustic impedances of the first and the second matching layers were calculated to be 9.32 and 2.37 MRayls for PZT-5H and 8.94 and 2.34 MRayls for LiNbO_3_. The first matching layer was the mixture of 2–3 μm silver particles (Sigma-Aldrich Co., Milwaukee, WI, USA) with Insulcast 501 and Insulcure 9 (American Safety Technologies, Roseland, NJ, USA). Parylene C (PDS2010, Specially Coating Systems Inc., Indianapolis, IN, USA) was selected as the second matching layer, which was also responsible for protection and electrical shielding. Both materials were chosen because their acoustic impedances were closest to the theoretical values among the materials available in our laboratory (see Table 1). The 105-MHz element was designed to have one acoustic matching layer that was Parylene C because it is difficult to control the thickness of the silver particle-based epoxy matching layers for high frequency ultrasound transducers [24]. The thickness of each layer was determined based on a PiezoCAD software package (Sonic Concept, Woodinville, WA, USA). The material properties and thickness of each layer used for the dual-element focused transducers are summarized in Table 1. From the PiezoCAD simulation, the center frequency and 6-dB fractional bandwidth of the 35-MHz element was expected to be 36 MHz and 57%, respectively, and those of the 105-MHz element were 107 MHz and 30% (Figure 4).

### 2.2. Transducer Fabrication

For fabrication of the 35-MHz element, a bulk PZT of 1.0 × 1.0 cm^2^ was fixed onto a glass plate using paraffin wax and lapped to a desired thickness of 59 μm. The lapped bulk PZT was sputtered with Cr/Au (500 Å/2000 Å) and then surrounded by plastic bars to construct dams. The mixture of 2–3 μm silver particles with Insulcast 501 and Insulcure 9, which is called silver epoxy in this paper, was poured into the dams on the bulk PZT, centrifuged, and cured at room temperature for 24 h in a dry box. The silver epoxy was lapped to a desired thickness of 11 μm and removed from the glass plate. The bulk acoustic stack was turned upside down on another glass plate and fixed again with paraffin wax. Backing material was cast onto the bulk PZT, cured at room temperature for 24 h, and lapped to a target thickness of 424 μm. The bulk acoustic stack was diced to a size of 0.5 × 0.5 mm^2^ using a dicing machine (DAD 322, Disco Corp., Tokyo, Japan). The 105-MHz element was comprised of one matching layer, LiNbO_3_, and a backing block. A bulk LiNbO_3_ of 1.0 × 1.0 cm^2^ was fixed onto a glass plate using paraffin wax and lapped to a desired thickness of 28 μm. The lapped bulk LiNbO_3_ was sputtered with Cr/Au (500 Å/2000 Å) and then surrounded by plastic bars to construct dams. The conductive epoxy was poured onto the bulk LiNbO_3_, cured at room temperature for 24 h, and lapped to a target thickness of 466 μm. This thickness was determined so that the final thickness of the 105-MHz element is equal to that of the 35-MHz element. The bulk stack piece was diced to a size of 0.5 × 0.5 mm^2^ using the dicing machine.

**Figure 5:**
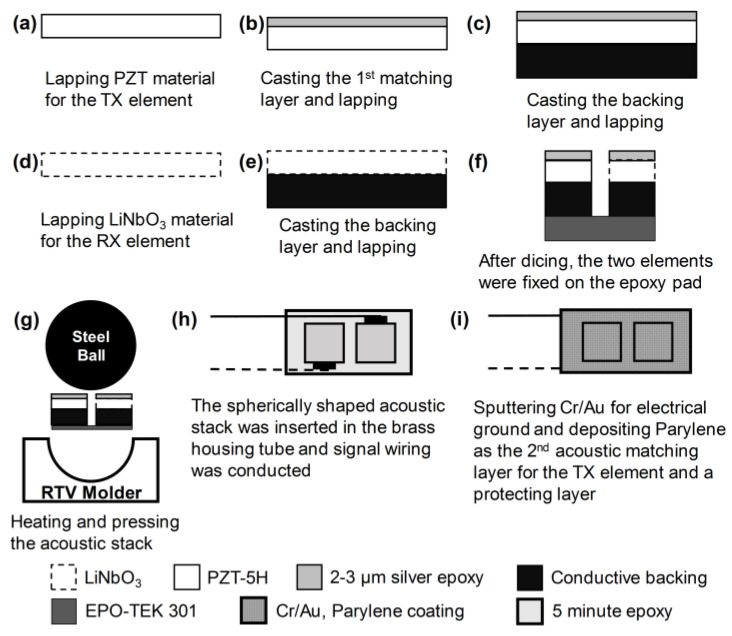
Illustration of the fabrication process for the dual-element focused transducer.

**Figure 6:**
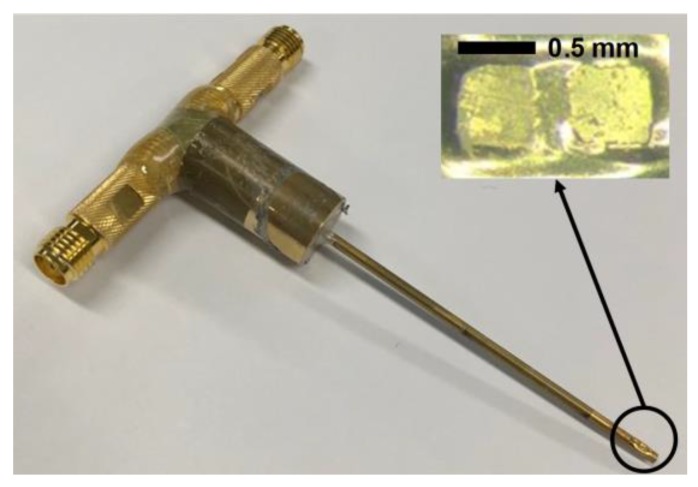
Photograph of the fabricated dual-element focused transducer for intravascular ultrasound tissue imaging using the third harmonic.

For geometric conformation of the two elements, an epoxy pad with a thickness of 200 μm was prepared. The epoxy pad was made of EPO-TEK 301 epoxy (Epoxy Tech Inc., Billerica, MA, USA), and its role was to prevent the connection between the electrical signal wires and ground. The two elements were fixed on the epoxy pad by using 5 min Epoxy (ITW Polymers Adhesive North America, Danvers, MA, USA), and press-focusing was performed using an RTV molder with a curvature of 2.5 mm radius for geometric focus. Details of the press-focusing method can be found in [25]. The spherically shaped acoustic stack was fixed with 5 min epoxy at the end of a brass housing tube with an inner diameter of 1 mm. For the electrical signal, two 42 AWG micro-coaxial cables were connected to the backing blocks of each element. The empty area in the brass housing was filled with 5 min epoxy for electrical insulation. Two signal wires were attached to a SMA connector used for connection to an imaging system. Cr/Au (500 Å/2000 Å) was deposited on the brass housing tube and the acoustic stack for ground connection. Finally, Parylene C was deposited for the first matching of the 105-MHz element as well as shielding of the transducer surface and the second matching of the 35-MHz element. Figure 5 illustrates the fabrication process, and Figure 6 shows the photograph of the fabricated dual-element focused transducer.

## 3. Performance Evaluation and Discussion

**Figure 7:**
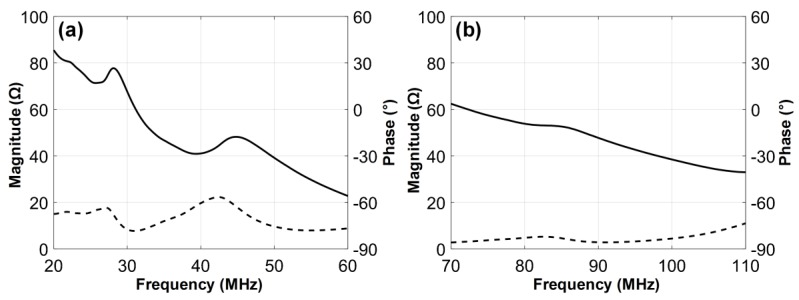
Measured electrical impedance magnitudes (solid line) and phase angles (dashed line): (**a**) 35-MHz and (**b**) 105-MHz elements of the dual-element focused transducer.

**Figure 8:**
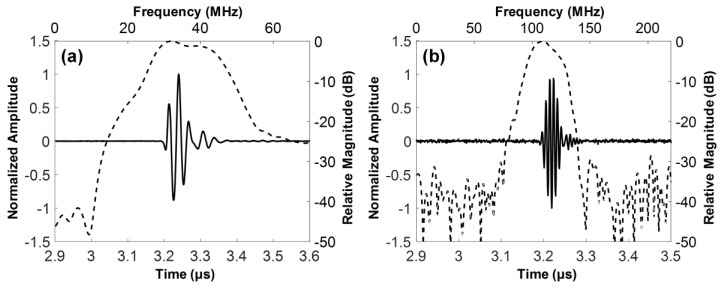
Measured pulse-echo responses (solid line) and their frequency spectra (dashed line): (**a**) 35-MHz and (**b**) 105-MHz elements of the dual-element focused transducer.

The electrical impedance of the fabricated dual-element focused transducer was measured using an impedance analyzer (HP4294A, Agilent Corp., Santa Clara, CA, USA) with z-probe attachment as shown in Figure 7. In the case of the 35-MHz element, the electrical impedance was measured at a magnitude of 46.7 Ω and a phase angle of −72.12° at 35 MHz. The 105-MHz element had the electrical impedance with a magnitude of 34.9 Ω and a phase angle of −79.85° at 105 MHz. Since a pulser/receiver system (UT340, UTEX Scientific Instruments Inc., Mississauga, ON, Canada) was used for pulse-echo tests and imaging performance evaluation, the electrical impedance of the 35-MHz element was considered to be well matched to the output impedance of the system, i.e., 50 Ω. In contrast, the 105-MHz element had the electrical impedance lower than the system’s output impedance, but this impedance mismatching was not a serious problem for operating the element. The problem was the phase distortion of the ultrasonic wave generated by the element, which led to elongating pulse length and thus degrading axial resolution. Therefore, we conducted electrical impedance matching using a series capacitor of 24 pF, a shunt capacitor of 13 pF, and a shunt inductor of 33 nH for minimizing the phase distortion. After the impedance matching, the electrical impedance magnitude and phase angle were changed to 34.9 Ω and −16.99°. For pulse-echo tests, the fabricated transducer was immersed into a container filled with a deionized water. A polished steel target playing a role of a reflector was placed in the container. The position of the transducer was adjusted using a motorized stage (SGSP26-100, SIGMAKOKI Co. Ltd., Tokyo, Japan) so that the focal point is located on the reflector. The pulser/receiver system was used to excite the elements and to receive the echoes from the reflector. The received echoes were recorded using an oscilloscope (DPO7054, Tektronics Inc., Beaverton, OR, USA) and used to calculate the center frequency and −6 dB fractional bandwidth of each element in MATLAB (Mathworks Inc., Natick, MA, USA). From the pulse-echo tests, it was found that the 35-MHz element had a center frequency of 36 MHz and a −6 dB fractional bandwidth of 56% (i.e., 26.1 to 46.6 MHz) as shown in Figure 8, which is in good agreement with the simulation results. However, the center frequency of the 105-MHz element was 112 MHz, which is higher than a prediction value of 107 MHz. In addition, the −6 dB fractional bandwidth was narrowed to 29% (i.e., 95.4 to 128 MHz). At the 105-MHz element, this discrepancy between the simulation results and the measurements may stem from the thickness of LiNbO_3_ being thinner than the target thickness (i.e., 28 μm). This is so because the lapping error leads to a considerable change in the center frequency of the 105-MHz element due to the very thin thickness of LiNbO_3_.

**Figure 9:**
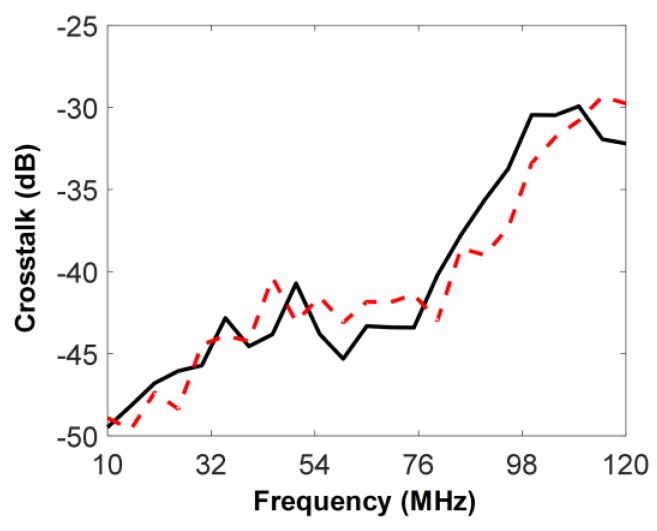
Measured crosstalk of the fabricated dual-element IVUS transducer. The solid line represents the crosstalk measured at the 105-MHz element while exciting the 35-MHz element. The dashed line indicates the crosstalk measured at the 35-MHz element while exciting the 105-MHz element.

The crosstalk between the two elements was measured. For this, the IVUS transducer was immersed into the water container, and the reflector was replaced by an acoustic absorber. A five-cycle sinusoidal burst signal with 5 V_p-p_ was generated by a function generator (AFG3102, Tektronix Inc., Beaverton, OR, USA) and was sent to both 35-MHz element and oscilloscope with an input impedance set to 1 MΩ. At the same time, the voltage induced by the sinusoidal signal at the 105-MHz element was measured using the same oscilloscope. The frequency of the sinusoidal signal was changed from 10 to 120 MHz at 5 MHz intervals and the ratio of the output voltage of the 105-MHz element to the input voltage of the 35-MHz element was computed (see the solid line in Figure 9); the maximum crosstalk was −29.9 dB at 110 MHz. In addition, the crosstalk was measured from the 35-MHz element while exciting the 105-MHz element (see the dashed line) and the maximum crosstalk in this case was −29.4 dB at 115 MHz. Note that the crosstalk measured in this study is a combination of acoustic and electrical crosstalk. At the design stage, acoustic crosstalk was expected to be ignored because the 5 min epoxy filled kerf for acoustically and electrically isolating the two elements had a relatively large value of 0.1 mm, compared to those of conventional arrays. However, it was anticipated that electrical crosstalk would dominate overall crosstalk because there were two parallel wires inside the brass housing with a diameter of 1 mm. This can be confirmed by the measured crosstalk that increases with the frequency used for the measurement as shown in Figure 9. This is so because a pair of coupled lines generate electrical crosstalk with each other, and the crosstalk is linearly proportional to frequencies below a few hundred MHz [26]. Nevertheless, the crosstalk of the fabricated dual-element IVUS transducer is acceptable for imaging because the crosstalk of conventional ultrasound arrays typically has a value between −28 and −32 dB [27].

**Figure 10:**
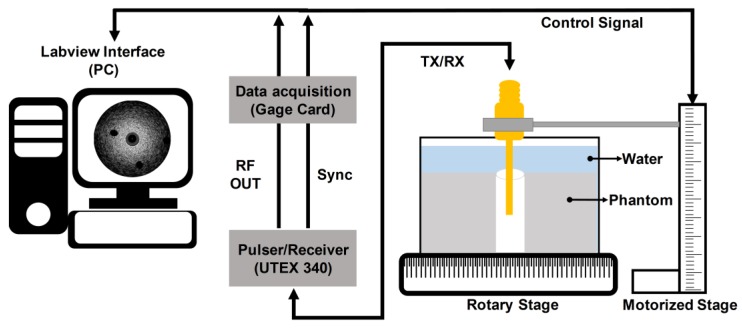
Schematic diagram of the experiment arrangement for the performance evaluation of the fabricated dual-element focused transducer.

The imaging performance of the developed transducer was also evaluated through tissue-mimicking phantom tests. For this, the tissue-mimicking phantom was constructed [28]; a deionized water of 250 mL in a beaker was heated to 60 °C on a hot plate. The mixture of an agar powder (A9799, Sigma-Aldrich Co. Ltd., St. Louis, MO, USA) of 8.0 g and a silicon dioxide powder (S5631, Sigma-Aldrich Co. Ltd., St. Louis, MO, USA) of 5.0 g were prepared and poured into the beaker while stirring the water with a rod. The mixture was cooled down at room temperature and cured in a refrigerator for 12 h. For insertion of the transducer, a hole was created in the center of the phantom. In addition, two 26 G 1/2 needles were used to create a lesion-mimicking area at the surface of the phantom; the diameter of each hole was 0.45 mm. To acquire image data, the developed transducer was inserted into the hole of the fabricated phantom in the deionized-water-filled container on the rotary stage (SGSP 160-YAW, SIGMAKOKI Co. Ltd., Tokyo, Japan) as shown in Figure 10. For the third harmonic imaging, the pulser/receiver system was used to excite the 35-MHz element and receive ultrasound by using the 105-MHz element. Note that 35- and 105-MHz fundamental images were also acquired using the pulser/receiver system. The received ultrasound was digitized and recorded using a Gage card (CS12502, Gage Applied Technologies Inc., Montreal, QC, Canada) in a personal computer. For one image, 1000 scanlines were acquired by rotating the stage in 0.36° increments. The scanning process was controlled by a program written in LabView (National Instrument, Austin, TX, USA). All signal processing for image construction with the recorded image data was conducted in MATLAB. For DC cancelling and noise reduction [29], a 129-tap band pass filter was used: cutoff frequencies of 21 and 52 MHz for the 35-MHz fundamental images and 90 and 133 MHz for both 105-MHz fundamental and third harmonic images. The images were logarithmically compressed with a dynamic range of 30 dB. Additionally, the spatial resolution of the fabricated transducer was measured using a 25-μm wire under the experimental setup. For this, the wire was placed at the focal point of the transducer, i.e., 2.5 mm before acquiring the 35-MHz fundamental, 105-MHz fundamental, and third harmonic scanlines. From the scanlines, the axial and lateral beam profiles were obtained after envelope detection [30], and their −6 dB beam widths were measured.

**Figure 11:**
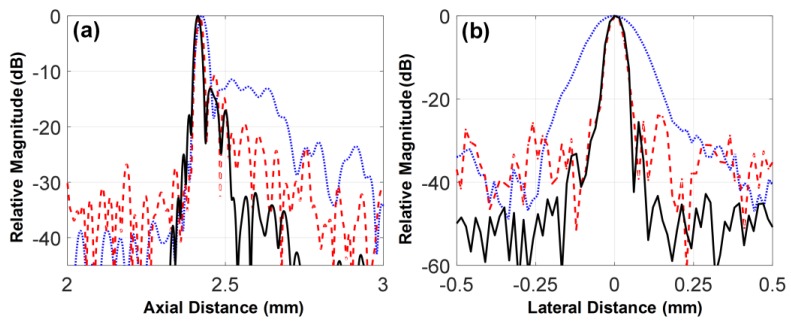
(**a**) Axial and (**b**) lateral beam profiles measured using a 25-μm wire located at the focal depth of the transducer, i.e., 2.5 mm. The dotted, dashed, and solid lines indicate the beam profiles obtained from the envelope signals of the 35-MHz fundamental, 105-MHz fundamental, and third harmonic scanlines, respectively.

As shown in Figure 11, the −6 dB axial and lateral beam widths of the 105-MHz fundamental and the third harmonic beam profiles were equal: an axial beam width of 25 μm and a lateral beam width of 46 μm. These were superior to the −6 dB axial and lateral beam widths of the 35-MHz fundamental beam profile, i.e., 40 and 153 μm, respectively. This is an obvious result because ultrasound frequency determines the widths of axial and lateral beam profiles directly related to the spatial resolution of ultrasound images. However, the level of side lobes associated with contrast resolution as well as spatial resolution was the lowest in the beam profile of the third harmonic, even though the beam profile was generated by the beam profile of the 35-MHz element that had the highest level of side lobes. This result demonstrates the superiority of the harmonic imaging in contrast resolution, which is possible because the energy of harmonics is nonlinearly proportional to transmitted ultrasound pressure and thus the main lobe energy in the beam profile of transmitted ultrasound mainly contributes to the generation of the harmonics.

**Figure 12:**
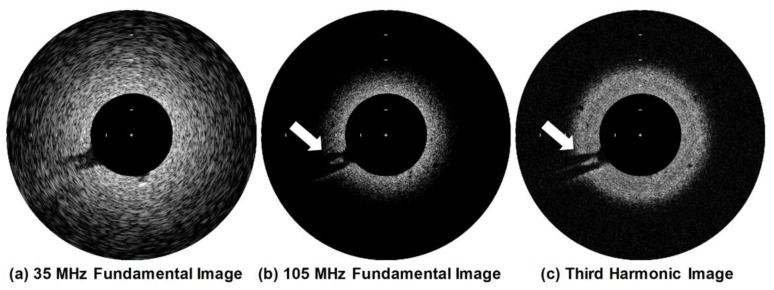
Ultrasound fundamental images of the tissue-mimicking phantom acquired by the (**a**) 35-MHz element and (**b**) 105-MHz element. (**c**) The third harmonic image was constructed using the image data acquired after transmitting ultrasound by the 35-MHz element and subsequently receiving the third harmonic signals generated in the phantom by using the 105-MHz element. The white arrows indicate the lesion-mimicking area. Logarithmic compression with a dynamic range of 30 dB was performed. The space between the white bars on the images indicates 1 mm.

Figure 12 shows the 35-MHz and 105-MHz fundamental images and the third harmonic image of the tissue-mimicking phantom. It was observed that the 35-MHz fundamental image had a deeper imaging depth than other images, but the speckle pattern appeared to spread along the arc as the imaging depth increased and the lesion-mimicking area in the phantom were not clearly seen due to low spatial resolution, compared to the others. In contrast, the 105-MHz fundamental image showed the conspicuous speckle pattern and clear lesion-mimicking area indicated by white arrows in Figure 12 due to its high spatial resolution. However, the frequency-dependent attenuation of ultrasound limited the imaging depth to less than 1.0 mm from the surface. The third harmonic image had a spatial resolution similar to the 105-MHz fundamental image, whereas its imaging depth was deeper than that of 105-MHz fundamental image, i.e., up to 1.4 mm. This result is reasonable because traveling distance of the fundamental ultrasound is twice the traveling distance of the third harmonic, so that the third harmonic experiences less attenuation.

**Figure 13:**
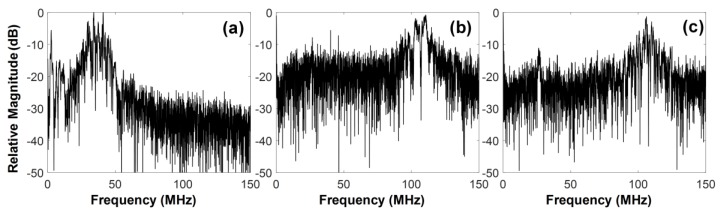
Frequency spectra of one scanline signal of the images in Figure 10: (**a**) 35-MHz and (**b**) 105-MHz fundamental imaging scanline, (**c**) third harmonic imaging scanline.

For tissue harmonic imaging, the spectrum of a transducer is typically divided into two sub-bands: one for transmission and another for harmonic reception. This causes lowering the axial resolution of both fundamental and harmonic images. Additionally, the complete separation of fundamental and harmonic signals is very challenging because their spectra are overlapped although pulse inversion and nonlinear chirp excitation can be used to overcome the problem partially [31,32,33]. Given these facts, the wide spectral bandwidth of a transducer is essential for high quality tissue harmonic imaging. However, it is practically impossible to construct a transducer with a broad bandwidth enough to carry out the third harmonic imaging of tissue. This is especially true for IVUS transducers because of their high operating frequency and limited selection of backing and acoustic matching materials due to the structural restriction. Even if possible, a severe degradation of spatial resolution is inevitable because the bandwidth should be divided into three sub-bands. As shown in Figure 13, the developed dual-element focused transducer can solve those problems. For ultrasound transmission, the full bandwidth of the 35-MHz element can be exploited, so that no degradation of spatial resolution occurs in fundamental images. This full-bandwidth signal contributes to the generation of the third harmonic, which is received by the 105-MHz element without (or at a minimum) loss of the harmonic information. Note that the frequency spectral characteristics of the 105-MHz element is similar to a band-pass filter, so that the signal separation is not necessary. Another advantage of the developed transducer is the simultaneous acquisition of co-registered 35- and 105-MHz fundamental images, which enables us to examine the overall morphological change of blood vessels and the near vessel layers such as fibrous cap with high spatial resolution at the same time if necessary.

## 4. Conclusions

In this paper, the 35-MHz/105-MHz dual-element focused transducer was designed and fabricated for intravascular ultrasound tissue imaging using the third harmonic. The characteristics of the developed transducer were examined and the imaging performance was evaluated using the custom tissue-mimicking phantom. Since the developed transducer allows the full-bandwidth fundamental and third harmonic imaging, decreasing spatial resolution, a common problem of tissue harmonic imaging, is no longer an issue. Due to this, the third harmonic imaging acquired by the developed transducer is capable of providing a deeper imaging depth than the 105-MHz fundamental imaging as well as spatial resolution similar to the 105-MHz fundamental imaging. As a further work, we will confirm the clinical usefulness of the proposed IVUS transducers through ex vivo and in vivo experiments on atherosclerosis lesion samples.

## Figures and Tables

**Table 1:** Material properties of piezoelectric materials, acoustic matching layers, and backing block used for the dual-element focused transducer.

Parameters	PZT-5H	LiNbO_3_	1st Matching	2nd Matching	Backing Layer
Longitudinal Velocity (m/s)	4700	7340	1900	2350	1850
Density (g/cm^3^)	7.8	4.65	3.86	1.1	3.2
Acoustic Impedance (MRayl)	36.7	34.1	7.334	2.59	5.92
Clamped dielectric constant	1200	27.9	-	-	-
Thickness of TX element (μm)	59	-	11	6	424
Thickness of RX element (μm)	-	28	6	-	466
